# HDAC6 dysfunction contributes to impaired maturation of adult neurogenesis in vivo: vital role on functional recovery after ischemic stroke

**DOI:** 10.1186/s12929-019-0521-1

**Published:** 2019-04-18

**Authors:** Joen-Rong Sheu, Cheng-Ying Hsieh, Thanasekaran Jayakumar, Guan-Yi Lin, Hsing-Ni Lee, Shin-Wei Huang, Chih-Hao Yang

**Affiliations:** 1 Department of Pharmacology, School of Medicine, College of Medicine, Taipei Medical University, No. 250, Wu Hsing St., Taipei, 110 Taiwan; 2 Graduate Institute of Medical Sciences, College of Medicine, Taipei Medical University, No. 250, Wu Hsing St., Taipei, 110 Taiwan

**Keywords:** Adult neurogenesis, Histone deacetylase 6, Ischemic stroke, Rehabilitation therapy, Retroviral labeling

## Abstract

**Background:**

Promoting post-stroke neurogenesis has long been proposed to be a therapeutic strategy for the enhancement of functional recovery after cerebral ischemic stroke. Despite numerous approaches have been widely reported the proliferation or differentiation of the neurogenic population therapeutic strategies by targeting adult neurogenesis not yet to be successfully clarified in clinical settings. Here, we hypothesized that alterations in microenvironment of the ischemic brain might impede the functional maturation of adult newly generated neurons that limits functional recovery after stroke.

**Methods:**

The in vivo retroviral based labeling model was applied to directly birth-date and trace the maturation process of adult newly generating neurons after hypoxic challenge. A rehabilitation therapy procedure was adopted through the combination of task-specific motor rehabilitating training with environmental enrichment to promote functional recovery after stroke. In addition, a pharmacological or genetic suppression of HDAC6 was performed to evaluate the functional significance of HDAC6 in the pathology of ischemic stroke induced deficits.

**Results:**

Serial morphological analyses at multiple stages along the maturation process showed significant retardation of the dendritic maturation on the newly generated neurons after stroke. Subsequent biochemical analyses revealed an aberrant nuclear translocation of HDAC6 that leads to the hyper-acetylation of α-tubulin (an indication of over-stabilized microtubules) after hypoxic challenge was observed at different time points after stroke. Furthermore, the mimicry experiments with either pharmacological or genetic suppression of HDAC6, phenocopied the stroke induced retardation in dendritic maturation of newly generating neurons in vivo. More importantly, we provide direct evidence showing the proper function of HDAC6 is required for rehabilitation therapy induced therapeutic benefits after stroke.

**Conclusion:**

Together, our current study unravels that dysfunction of HDAC6 contributes to stroke induced deficits in neurogenesis and provides an innovative therapeutic strategy that targets HDAC6 for promoting functional recovery toward the patients with stroke in clinic.

## Introduction

Ischemic stroke is one of the leading life-threatening neuropathology, which causes millions of deaths and long-term disability worldwide. Except of thrombolytic agent: recombinant tissue plasminogen activator (rt-PA) for the removal of thrombus at the acute phase of ischemic challenge, there is no other established treatment to reduce the neurological deficits caused by ischemic stroke. Therefore, there is an urgent need to develop innovative and efficient therapeutic strategies toward promoting functional recovery after hypoxia challenge. Despite several considerations that enhance neurogenesis after ischemic stroke have been widely investigated [1–3], however, so far such therapeutic approaches have yet led to successful clinical outcomes.

Adult neurogenesis was conventionally believed to take place only during embryonic development in mammals [4]. Only recently has it become generally accepted that new neurons are continuously produced in isolated regions of the adult mammalian CNS that it actively occur throughout the whole life in the subventricular zone and the subgranular zone of the dentate gyrus in the hippocampus [5–7]. Through a dedicated maturation process, these newly generated neurons gradually integrated into the existing neural circuitry and actively contribute to critical roles in certain cognitive functions, such as spatial memory storage and actions of antidepressants in the adult brain [8, 9].

As a potential hope for the treatment of stroke, numerous studies have examined the functional significance of adult neurogenesis on post-stroke neurological performance. It has been reported that ablating adult neurogenesis results in impaired functional recovery and exacerbation of neurological deficits which indicated a positive therapeutic potential for the newly generated neurons in the repair of the damaged brain [10, 11]. On the contrary, controversial evidence showing that increase in the number of adult newly generated neurons by voluntary running or pharmacological stimulator of neurogenesis after stroke exacerbated individual severity in cognitive impairment [12], which suggested an adverse impact of these newly generated neurons. Based on this previous controversial evidence, we raised the hypothesis that the microenvironment of the brain might not be suitable for the proper functional maturation of the newly generating neurons after stroke. And the aberrant growing of these newly generating neurons might impede the functional recovery of the brain after hypoxic challenge. Although few pioneering studies have provided evidences showing the changes in morphology of newborn neurons after MCAO [13, 14], these reports are merely focused on a single time point without detail analyses along the maturation process of these newly generating cells. Besides, the extent and duration of the deleterious influence of a hypoxic event on the proper growing of these cells have yet been checked carefully. More importantly, the possible pathological mechanisms and therapeutic strategy for targeting of these aberrant cells for promoting functional recovery after hypoxic challenge are still largely unknown.

As the major cytoskeletal component of neurites, proper elaboration of dendritic arborization depends on microtubules for functional and structural support [15]. Microtubules are cylindrical cytoskeletal structures, which undergo a dynamic process of assembly and disassembly that control the flexible cell shape remodeling, cell motility, tubulin stability and terminal branching of dendritic arborization in neurons. It has reported that reversible acetylation on Lys40 of α-tubulin marks stabilized microtubule structures and has been found to be crucial in regulating of microtubule dynamics [16]. HDAC6 (histone deacetylase 6) which is the major cytoplasmic α-tubulin deacetylase [17], regulates the dynamic flexibility of the microtubules through the enzymatic deacetylation ofα-tubulin in assembled microtubules. Accumulating evidence indicated that HDAC6 dysfunction leads to hyper-acetylation of α-tubulin that impedes the dynamic structural remodeling of microtubules.

Here in our current study, through the application of in vivo retroviral based labeling approach to directly birth-date and comprehensively evaluate the maturation process of adult neurogenesis after hypoxic challenge provide evidence showing a long lasting deleterious influence of hypoxia on dendritic maturation of adult neurogenesis. Meanwhile, the aberrant nuclear translocation of HDAC6 and the loss in microtubule dynamic regulation contributes to the observed phenotypes. Pharmacological and genetic experiments further emphasized the requirement of HDAC6 in rehabilitation therapy induced therapeutic benefits after stroke.

## Material and methods

### Materials

ACY-738 was purchased from Selleckchem (Houston, TX, USA) and prepared as stock solution with the concentration of 50 mg/ml by DMSO. Tubastatin A was obtained from Cayman Chemical (Ann Arbor, MI, USA) and prepared as stock solution with the concentration of 10 mg/ml by DMSO. The stocks were further diluted by 0.9% saline solution before injection in the animals. DAPI, Dimethyl sulfoxide (DMSO) and bovine serum albumin (BSA) were purchased from Sigma-Aldrich (St. Louis, MO, USA). Primary antibodies against Acetyl-α-Tubulin at Lys40 (mAb 5335) and anti-α-Tubulin (mAb 3873) were purchased from Cell Signaling (Beverly, MA, USA). HDAC6 antibody (H-300) was from Santa Cruz Biotechnology (Santa Cruz, CA, USA), β-actin antibody (MA5–11869) was purchased from Thermo Fisher Scientific.

### Animals

Animal experiments were conducted using six to ten week-old C57BL/6 mice of both sexes (BioLASCO, Taipei, Taiwan). All the experimental procedures for animal handling or surgeries were approved by the Institutional Animal Care and Use Committee (IACUC) of Taipei Medical University (TMU) and were in accordance with the guide for the Care and Use of Laboratory Animals. All animals were reared in a temperature-controlled environment maintained on a 12 h light/dark cycle.

### Middle cerebral artery occlusion (MCAO) surgery in mice

The protocol of MCAO surgery for mice is basically conformed to the Guide for the Care and Use of Laboratory Animals (NIH publication no. 85–23, 1996) and was approved by IACUC of TMU. For the MCAO surgery, mice were anesthetized with a mixture containing 75% air and 3% isoflurane maintained in 25% of oxygen. Mice were placed on a 37 °C heating pad throughout and after the surgery for another two hour for maintaining the body temperature at 37 °C ± 0.5 °C. For the procedure of transient MCAO, the right common carotid artery (CCA) was exposed and a 6–0 monofilament nylon suture (20 mm) coated with silicon (3 mm) was inserted from the external to the internal carotid artery until the tip occluded the right middle cerebral artery (MCA) which is approximately 10 mm from the surgical incision site [18]. Then, the filament suture was kept for the occlusion of MCA for thirty- or sixty-minutes and then gently withdrawal to regain the blood reperfusion for the mice.

### Construction, production, and stereotaxic injection of engineered retroviruses

High titers of engineered self-inactivating murine retroviruses (1 × 10^9^ unit/ml) expressing green fluorescent proteins (GFP) were produced for the labeling and trace the newly generated neurons in the adult dentate gyrus. To obtain a more stable and reliable expression of the GFP protein for the visualization of dendritic morphology, the green fluorescent protein expression plasmid was constructed under the control of ubiquitin promoter. And a retroviral vector with β-actin fused GFP was also constructed for experiments with morphological analyses of dendritic spines in vivo for the better visualize of the detail structure of the protrusions. For the experiments with genetic silencing of HDAC6 cell autonomously in the newly generated neurons, two validated (short-hairpin RNAs) shRNA that genetic silencing of HDAC6 [19] and one control shRNA (shDsRed) were constructed into the retroviral vector. The targeting sequences the silencing of HDAC6 is GCACAATCTTATGGATGGGTA for shHDAC6-a and CCTTGCTGGTGGCCGTATTAT for shHDAC6-b.

Retroviral particles were prepared by co-transfection of the viral expression plasmid along with the helper plasmid into the HEK-293 cells. And then medium was collected at 48 h after transfection for the ultracentrifugation to concentrate the recombinant pseudo-viral particles [20]. The concentrated retroviruses were stereotaxically injected at four sites in the dentate gyrus (0.5 μl per site at a speed of 0.25 μl / min) with the following coordinates: anterioposterior = − 2 mm from bregma; mediolateral = ± 1.6 mm and dorsoventral = 2.5 mm; anterioposterior = − 3 mm from bregma; mediolateral = ± 2.6 mm and dorsoventral = 3.2 mm. To avoid the back flow of the viral solution, the injection needle was kept in place at the site of injection for an additional 5 min before being slowly removed.

### Tissue sectioning, imaging, and quantification

Mice were deeply anesthetized with the air mixture containing 75% air and 5% isoflurane maintained in 25% of oxygen and perfused transcardially with phosphate-buffered solution followed by 4% formaldehyde. And then the brain was removed and incubated for fixation in 4% formaldehyde for one night and transferred to a 30% sucrose solution and stored at 4 °C until sectioning. Coronal brain sections in 60 μm were prepared by the sliding Microtome (Leica, Nussloch, Germany) and the sections containing the entire the hippocampal dentate gyrus were used for the imaging. The sections were washed three times with phosphate-buffered solution and then incubated with 0.1 μg/ml of 4′,6-diamidino-2-phenylindole (DAPI) in PBS solution for the counterstaining of the granular cell layer of the dentate gyrus. And then the slices were mounted and covered with the mounting solution (Vector Laboratories) on a glass coverslip. Images were obtained under the Leica TCS SP5 confocal spectral microscope imaging system by using an argon or krypton laser through the 40x, 1.40 NA oil immersion objective (Mannheim, Germany). Three-dimensional reconstructions of the dendritic morphology of the newly generated neurons were processed by the software Imaris and further analyzed for the total dendritic length or the number of branches of individual neurons by the Neuron J plugin of the software Image J (NIH, USA).

### Rehabilitation therapy

Since the upper limb disability is a common problem in post-stroke patients, the task-specific training is regarded as the most reliable rehabilitation therapy in clinic. But the therapeutic benefits of neurorehabilitation training usually does not generalize to neurological improvement other than the ones trained, so we applied a protocol with the combination of both task-specific motor rehabilitation therapy with cognitive intensive stimulus of environmental enrichment. For the task-specific forelimb rehabilitation training, the mice were trained to reach and grasp for a sucrose pellet placed outside the home cage at a specified distance. After the animals got familiar with the testing environment, the sucrose pellet was moved to the left side which forced the animals to use the right (later been affected by stroke) forelimb to retrieve the sucrose pellet. After five days of habitation training, most of the animals could learn the motor task and reach a plateau performance in more than 50% of the trials. And then, animals were conducted with the MCAO surgery and received the rehabilitation therapy at seven days after hypoxic challenge. During the rehabilitation therapy, mice were given 50 training trials per day for two weeks. Meanwhile, the cognitive intensive stimulus of environmental enrichment of the home cage was combined in our protocol of rehabilitation therapy to provide synergistic functional recovery of the brain. Basically, the animals were provided with sensory stimulatory toys or novel objects in different shape during the two week period of rehabilitative training, and we randomly changed the novel objects every two days to make sure the high levels of cognitive and sensory stimulation of our rehabilitation treatment.

### Behavioral analyses-adhesive tape removal test

For the sticky tape test, two strips of tape (3 × 4 mm) with the shape in rectangular were applied with equal pressure on the hairless part of both forepaws. The time for the animals spent to contact (reflect of sensory function) and remove (reflect the motor performance) the tape on both forepaws (left and right) was recorded. The animals were given three days habituation training (two training trial per day) before the MCAO surgery and then measured their time-to-contact and the time-to-remove the tapes at different time points after the MCAO surgery. A maximum duration of 120 s was given for the mouse to conduct the adhesive removal test.

### Behavioral analyses-object location memory task

The experimental apparatus for OLM test was composed of a white-color box with 40 × 40 × 40 cm^3^ in its size and placed in the dimmed light-illuminated room. The animals was given a ten-minute habitation phase to explore the box without any testing objects before the experiments, and different visual cues were attached on the walls of the testing apparatus to provide contextual guiding information. During the training phase, the testing mouse was placed in the testing apparatus and was allowed to explore two identical objects located at two diagonal corners for ten minutes and returned to their home cage. Thirty-minutes later, the testing mouse was placed in the testing apparatus with one of the object been repositioned to another corner of the box. The testing phase consisted of five minutes exploration of the two objects, and the time spent in exploring each object was recorded using a digital video camera and scoring for the preference for the repositioned object by the behavioral tracking system Ethovision XT (Noldus). A discrimination index was calculated by the formula of [time spent on the object in novel location / (time spent on the object in novel location + time spent on the object in familiar location)] × 100% to reflect the cognitive performance.

### Behavioral analyses-Rotarod activity test

Before the evaluation of motor coordination by the rotarod test, mice were first trained daily for a consecutive three days for the habituation of the task. Individual animal were trained to run on an accelerating rotating rod (speed up from 4 to 40 rpm over three minutes with increasing steps of 6 rpm every 30s). Before and at different time points (from one to twenty-eight days) after the MCAO surgery, the mice were placed on the rotarod and assessed for their body balance and motor coordination (UGO Basile, Varese, Italy) over a period of maximum five minutes. The time (in seconds) for each of the individual mouse fell from the drum will be recorded for up to 180 s through the automatic stopwatch.

### Pharmacologic experiments with HDAC6 inhibitors

For the experiments with pharmacologic suppression of HDAC6, Tubastatin A (a highly selective HDAC6 inhibitor, 0.5 mg/kg) or ACY-738 (a HDAC6 inhibitor with high blood-brain-barrier permeability, 5 mg/kg) or vehicle solvent (0.9% saline solution) were intraperitoneally injected every two days for the mice. The changes in body weight of individual animals were recorded daily to make sure there is no acute drug related toxicity for the animals.

### Immunoblotting

For the biochemical analyses of molecular events linked to changes in the adult newly generated neurons from the brain, we first micro-dissected the granule cell layer of the dentate gyrus where the active neurogenesis take place in the adult brain. A population of mice were given the transient MCAO surgery, and sacrificed at different time points after the MCAO. Coronal brain sections at 1 mm thickness which contained the hippocampal dentate gyrus were prepared and the granule cell layer of the dentate gyrus was surgically dissected under the dissecting microscope. Tissues were homogenized and sonicated in an ice-cold Tris-HCl lysis buffer solution, pH 7.4, containing a mixture of protein phosphatase and proteinase inhibitors and centrifuged at 12, 000×*g* for 20 min at 4 °C. Samples of equal amounts of proteins were subjected to 8 or 10% SDS-PAGE, and the proteins were electro-transferred to the PVDF membranes using a Bio-Rad semi dry transfer unit (Hercules, CA, USA). Then the membrane was blocked with 5% (*w*/*v*) non-fat milk in TBST (10 mM Tris-base, 100 mM NaCl, and 0.02% Tween 20) for an hour and probed with primary antibodies overnight at 4 °C. The concentration of primary antibodies are used as: Acetyl-α-Tubulin and α-Tubulin (1:10000), β-actin (1:5000), HDAC6 (1:500) and Lamin B1 (1:1000). After extensive wash with TBST, the membranes were incubated with the appropriate HRP-linked secondary antibody. Then, the immunoreactive bands were visualized with enhanced chemiluminescent reagents (ECL, Amersham, UK) by the scientific imaging system (Biospectrum AC System, UVP) and analyzed by the plugins of Image J.

### Separation of cytoplasmic and nuclear fractions

Twenty mg of micro-dissected tissue blocks were used for the isolation of cytoplasmic and nuclear fractions by using the NE-PER Extraction Reagents (Thermo Fisher Scientific, USA) according to the manufacturer’s instructions. Tissue was first homogenized for the disruption of cell membrane and release of cytoplasmic contents, then the centrifuged pellets which contain the nuclei were further extracted to obtain the nuclear proteins. Lamin B1 and α-tubulin were used as internal controls for the nucleus and cytosol, respectively.

### Statistical analysis

All the results are presented as means ± SEM. The significance of any difference between two groups was calculated using the two-tailed unpaired student *t*-test. The comparison between multiple groups of results were analyzed with one-way or two-way analyses of variance (ANOVAs) followed by Bonferroni’s post hoc analyses for evaluating the significance between isolated groups. Correlation analyses between two parameters were performed using Pearson’s correlation analysis. All the statistical analyses were performed using the software of Prism6 (GraphPad Software). Probability values of *p* < 0.05 were considered as significant differences.

## Results

### Retroviral labelling of newly generated neurons after MCAO

**Fig. 1:**
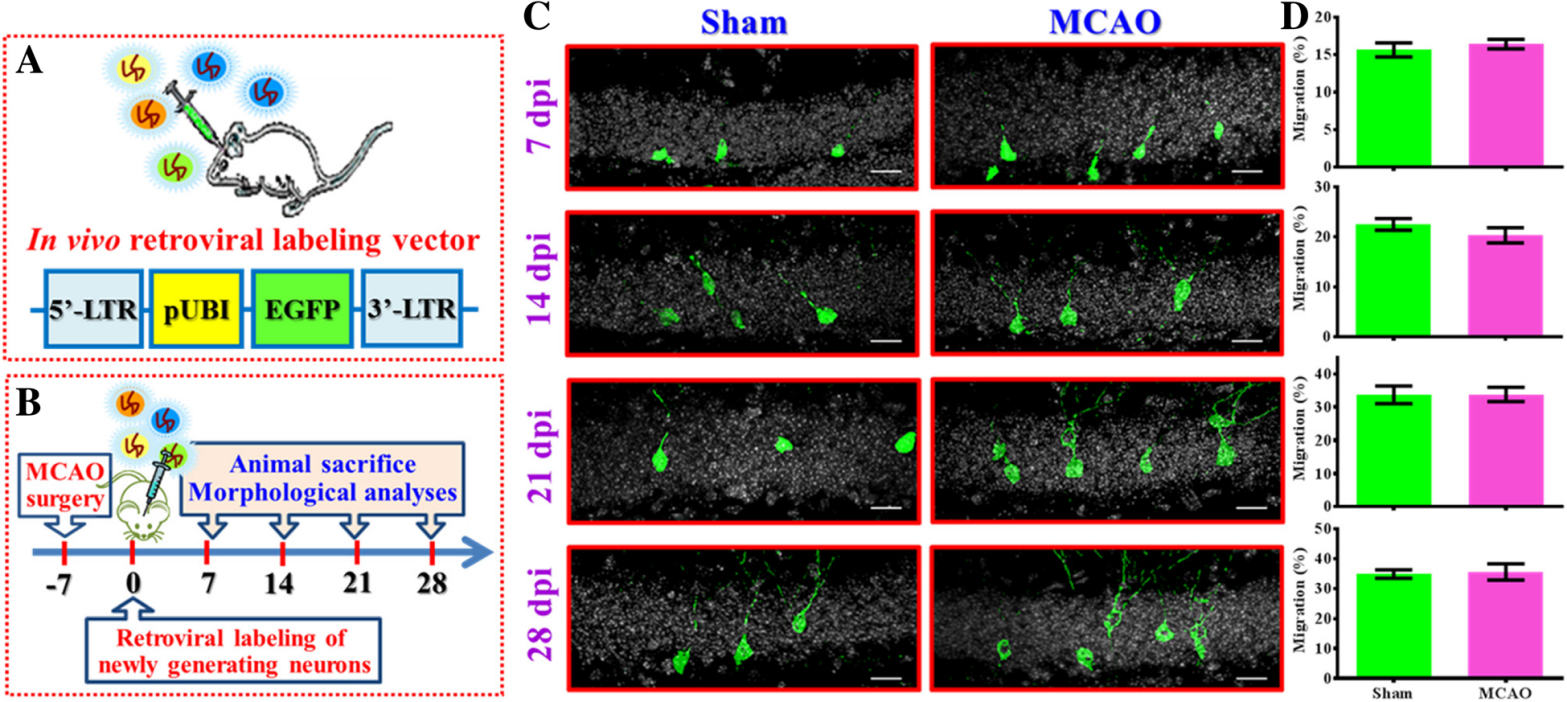
Retroviral labeling of newly generated neurons after stroke. (**a**) The retroviral vector used in the study for labeling and tracing the newly generated neurons after MCAO surgery in vivo. LTR: Long terminal repeats, pUBI: Ubiquitin promoter, EGFP: enhanced green fluorescent protein. (**b**) Schematic illustration of the procedure of experimental designs. (**c**) Representative images of the retroviral labeled newly generated neurons obtained at 7, 14, 21 or 28 days-post viral-injection (dpi). Green, GFP signal from labeled newborn neurons. White, DAPI signal for the granular cell layer. The scale bar is 25 μm. (**d**) Quantification of the relative migratory distribution of the soma from the labeled cells along the granular cell layer at 7, 14, 21 or 28 dpi. Data are presented as mean ± SEM. *n* = 30 to 50 cells from four mice, two-tailed unpaired *t*-test

To visualize the maturation of adult newly generated neurons, a retroviral-based single cell labeling approach was used **(**Fig. 1a**)**. Besides, a thirty-minute MCAO was used to make sure the core lesion area is restricted just to the cortex without a direct traumatic damage of the hippocampal neurogenic area. Experimentally, six-week old C57BL/6 mice were challenged with transient MCAO surgery. One week later, retroviruses were stereotaxically injected into the dentate gyrus for labeling of the newly generating neurons born at one week after hypoxic challenge. And then, the animals were sacrificed at different time points after retroviral injection (7, 14, 21 or 28 days-post-injection (dpi) for a comprehensive visualization of the maturation of the newly generating neurons in the hypoxic brain (Fig. 1b). High-resolution three-dimensional reconstructions of the neuronal morphology were imaged by laser scanning confocal microscopy for the evaluation of proper maturation of newly generating neurons at different precise developmental time points. Variety of morphological indexes including the migration of the neuronal soma, the total dendritic length, the complexity of dendritic tress were analyzed to reflect the neuronal maturation process.

By analyzing the relative distribution of the soma of individual newly generating neurons along the granular cell layer of the dentate gyrus (Fig. 1c), we first analyzed the migratory phenotypes of these newly generated neurons in the hypoxic brain. After generating from the neurogenic niche along the edge of the hilus, newly generating neurons start migrating and distributed gradually along the granular cell layer. We found the newly generated neurons grow in the brain of sham-operated control animals migrated gradually from inner granular cell layer to the outer regions at different time points after labeling (Fig. 1, c-d 7 dpi: 15.65 ± 0.9747%, 14 dpi: 22.52 ± 1.172%, 21 dpi: 33.73 ± 2.651%, 28 dpi: 34.9 ± 1.433%). When compared with the neurons grow in sham-operated animals, we found the newly generated neurons grow in the hypoxic brain displayed similar migratory behaviors along the granular cell layer at different time points after labeling (Fig. 1c-d, 7 dpi: 16.43 ± 0.638%, 14 dpi: 20.31 ± 1.526%, 21 dpi: 33.84 ± 2.177%, 28 dpi: 35.61 ± 2.719% for MCAO group). And, there is no significant difference in the migratory phenotypes of these newly generated neurons between two groups along the developmental time points we tested.

### Aberrant maturation of newly generated neurons in the hippocampus after MCAO

**Fig. 2:**
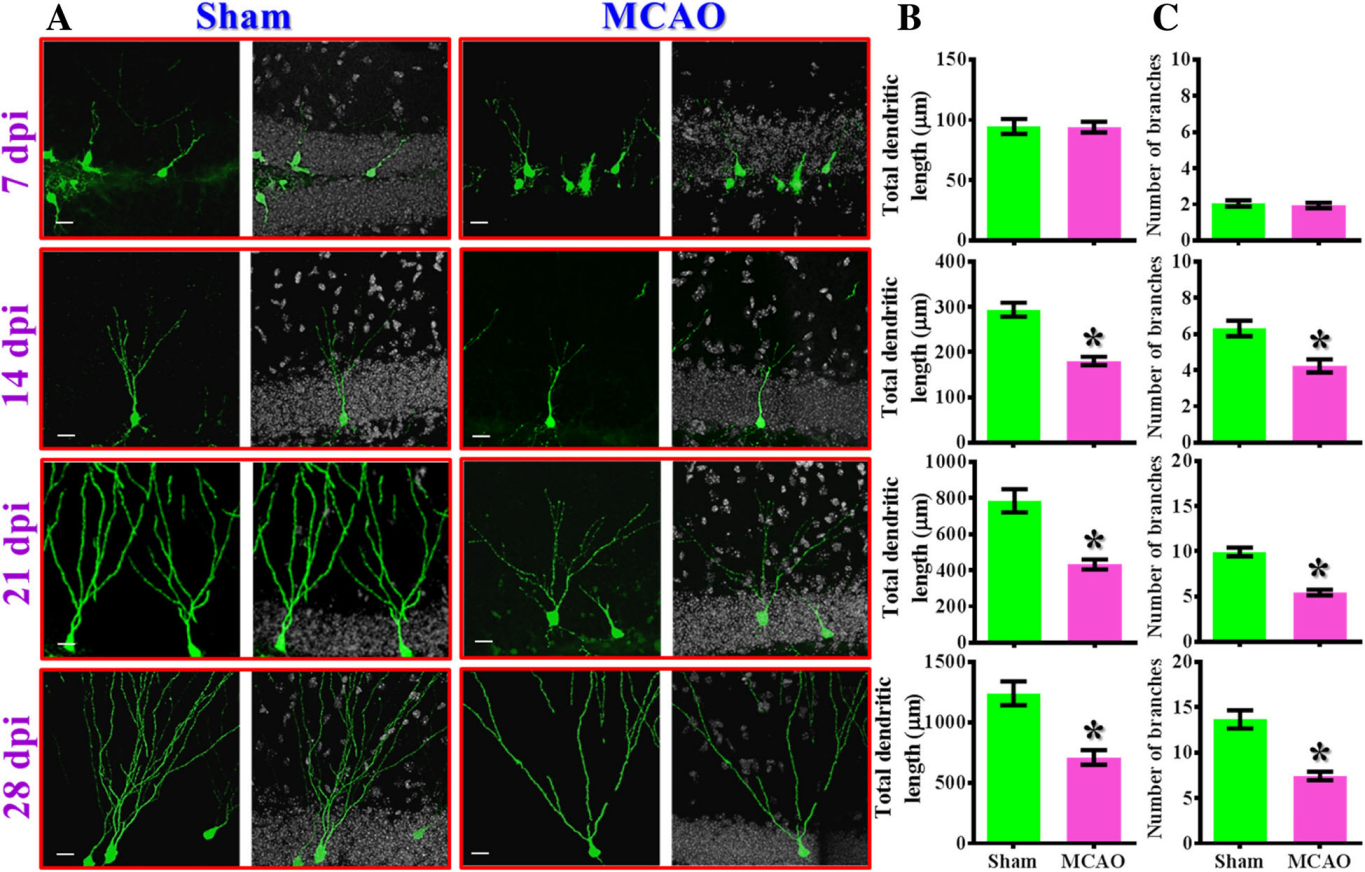
Aberrant maturation of newly generated neurons in the hippocampus after MCAO. (**a**) Representative images of the retroviral labeled newly generated neurons born at seven days after MCAO surgery and imaged for their dendritic elaboration at 7, 14, 21 or 28 dpi. The scale bar is 25 μm. (**b**) Quantification of total dendritic length of individual newborn neurons obtained from 7, 14, 21 or 28 dpi. (**c**) Quantification of the number of dendritic branches from individual newborn neurons obtained from 7, 14, 21 or 28 dpi. Data are presented as mean ± SEM. *n* = 20 to 40 cells from four mice, two-tailed unpaired *t*-test. * *p* < 0.05 compared to sham-operated control

As the proper elaboration of dendritic branching is crucial for the new neurons to integrate into the existing circuits and receive input information, we then examined if the proper elaboration of dendritic trees might be affected after the MCAO surgery. We found the newly generating neurons radially extend their dendrites and form complex branching of dendritic structures along the maturation process in both sham-operated or MCAO animals (Fig. 2a). However, we found there is a significant (*p* < 0.05) reduction in the total dendritic length (Fig. 2b**)** and the number of dendritic branches **(**Fig. 2c**)** for the time points of 14, 21, and 28 dpi from MCAO group vs. sham-operated control for the newly generating neurons grow in the hypoxic brain. These data suggested that hypoxic challenge indeed impede the morphological maturation process of the newly generating neurons.

### MCAO alters the maturation of dendritic spines of newborn neurons

**Fig. 3:**
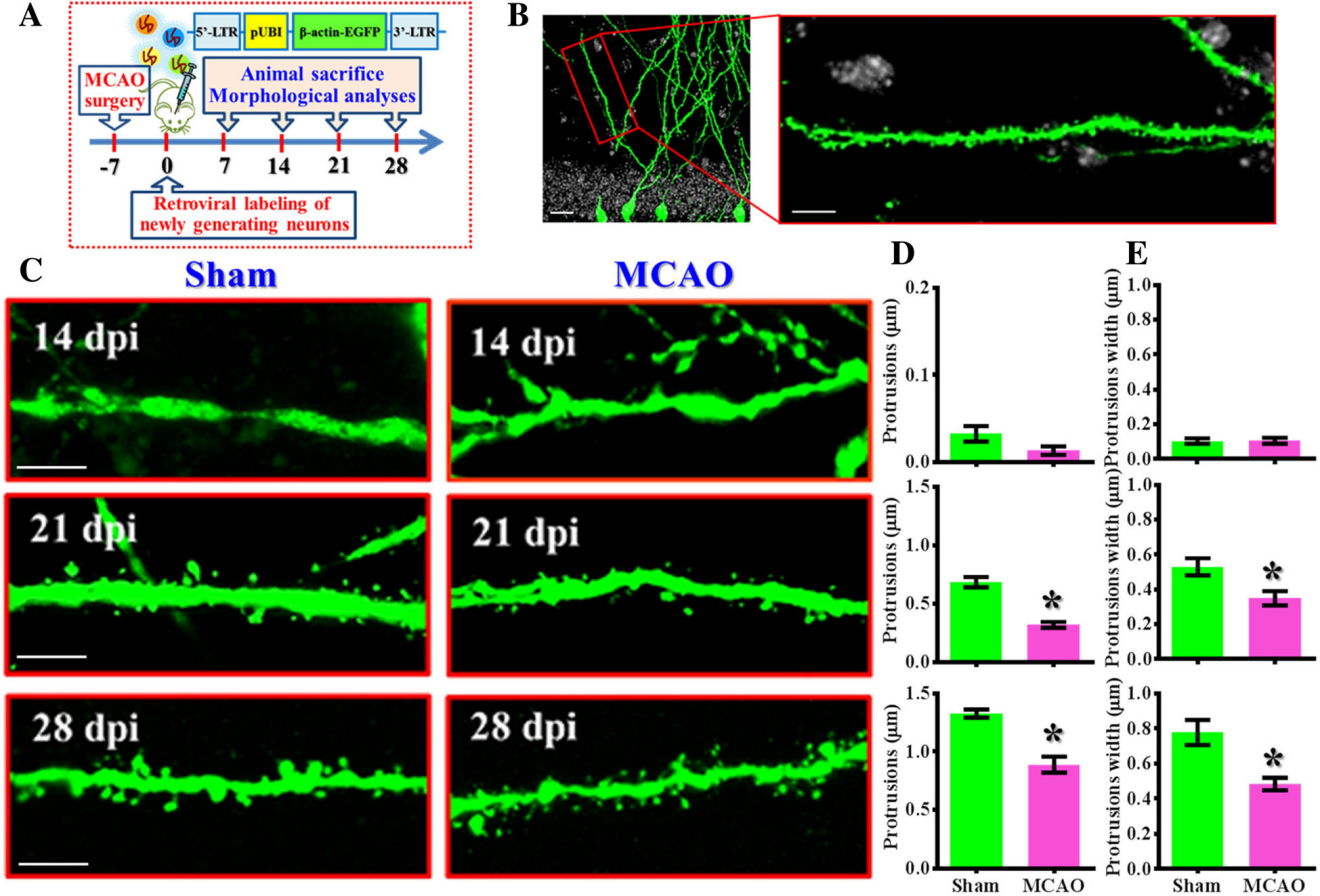
Alteration in maturation of dendritic spines of newborn neurons after MCAO. (**a**) Schematic illustration of the procedure for the visualization of maturation in dendritic protrusions in vivo after stroke. EGFP fused with β-actin protein was used for their preferential localization in dendritic protrusions. (**b**) Representative image and its enlarged inset showing the selection of an isolated dendritic branch located at 50 to 100 μm from the neuronal soma for the analyzing of dendritic protrusions. The scale bar is 10 μm. (**c**) Representative images of the dendritic protrusions from the retroviral labeled newly generated neurons born at seven days after MCAO surgery and imaged at 14, 21 or 28 dpi. The scale bar is 5 μm. (**d**) Quantification of the number of dendritic protrusions per μm of dendritic tree obtained from 14, 21 or 28 dpi. (**e**) Quantification of the protrusion width of individual dendritic spines obtained from 14, 21 or 28 after viral injection. Data are presented as mean ± SEM. n = 20 to 40 dendrites from three to four mice, two-tailed unpaired *t*-test. * *p* < 0.05 compared to sham-operated control

Dendritic spines, the bulbous protrusions that serve as the connecting points between two neuronal cells, are the most prominent features to reflect functional maturation of the newly generated neurons. Next, we analyzed the morphology of the dendritic spines to see the impact of hypoxic challenge on the maturation process of the newly generating neurons. Retroviral particles expressing β-actin fused GFP were stereotaxically injected into the dentate gyrus one week after MCAO surgery (Fig. 3a). And then animals were sacrificed at 14, 21 or 28 days after retroviral labeling for visualization of the dendritic protrusions (Fig. 3b). We found an increasing number of protrusions and the width of individual protrusion of newly generating neurons along the maturation process in sham-operated control animals (Fig. 3c-d) suggesting the functional maturation of these cells. However, the newly generated neurons grow in the hypoxic brain displayed significant (*p* < 0.05) reduction in the number of protrusions (Fig. 3c-d**)** and immature phenotypes of their protrusions width (Fig. 3c and e**)** for the time points of 21 and 28 dpi from MCAO group vs. sham-operated control. Together, through the in vivo retroviral based labeling approach to trace the maturation process of adult newly generated neurons after hypoxic challenge, we provide comprehensive evidence showing the aberrant maturation of newly generating neurons in the hippocampus after the hypoxic challenge.

### Lasting deleterious impact of a single event of hypoxic challenge on the maturation of newly generating neurons

Our findings of aberrant neuronal maturation in the ischemic brain raised another important question: how long would be the deleterious influence lasted after the experience of a single event of hypoxic challenge? To answer this, morphological maturation of newly generating neurons were examined by different intervals between the retroviral labeling of their birth with the exposure of hypoxic event (7-, 14-, 21- or 28-days post-stroke). Animals were sacrificed at fourteen days after retroviral injection (14 dpi) for the visualization of morphological maturation of these cells.

**Fig. 4:**
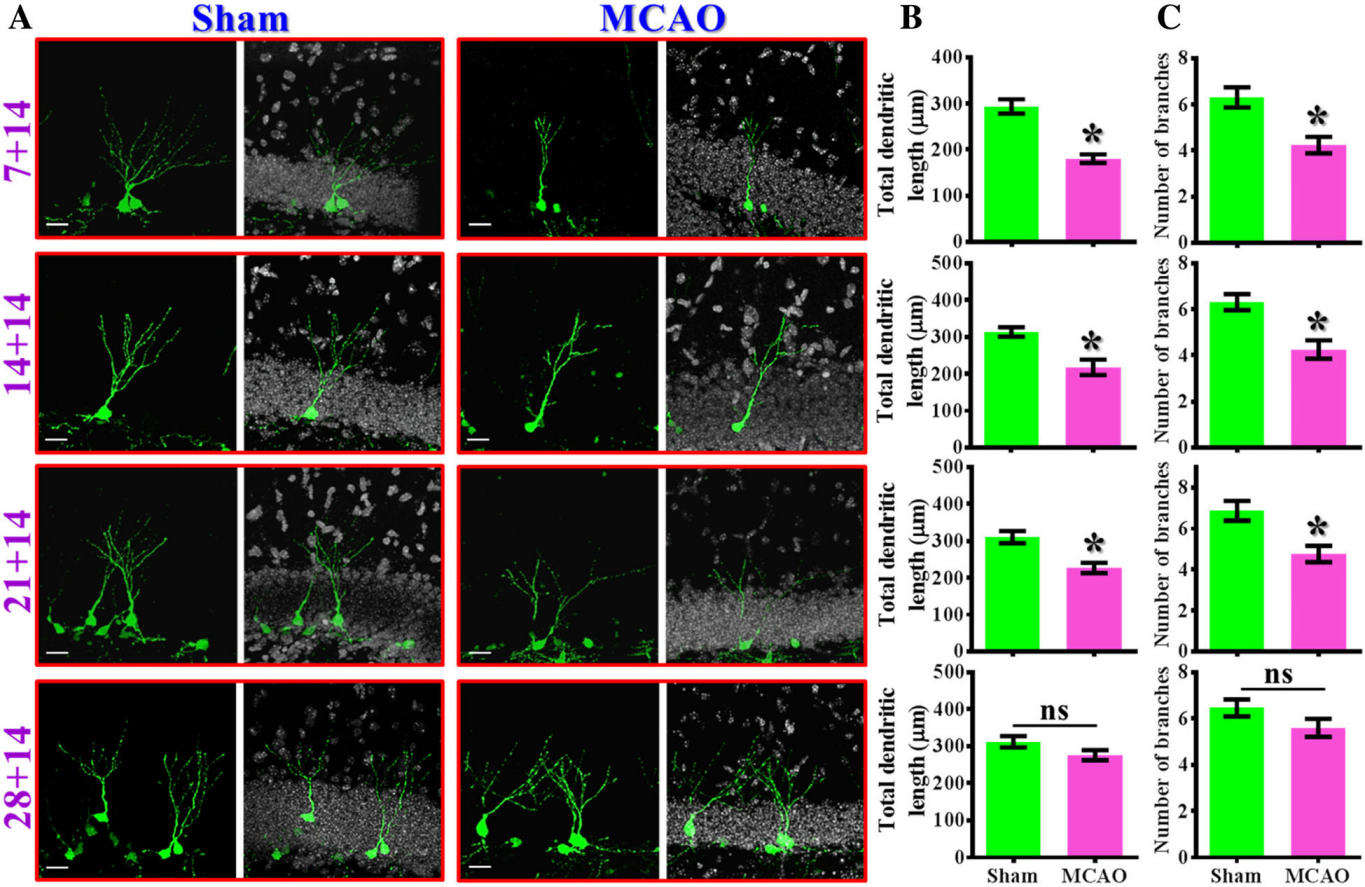
Lasting deleterious influence on dendritic maturation after hypoxic challenge. (**a**) Representative images of the retroviral labeled newly generated neurons born at different time points (7, 14, 21 or 28 days) after MCAO surgery and imaged for their dendritic elaboration at 14dpi. The scale bar is 25 μm. (**b**) Quantification of total dendritic length of individual 14 dpi newborn neurons born at 7, 14, 21 or 28 days after MCAO surgery. (**c**) Quantification of the number of dendritic branches from individual 14 dpi newborn neurons born at 7, 14, 21 or 28 days after MCAO surgery. Data are presented as mean ± SEM. *n* = 20 to 40 cells from four mice, two-tailed unpaired t-test. * *p* < 0.05 compared to sham-operated control. “ns” represents no significant

A significant reduction in the total dendritic length (Fig. 4a and b, *p*< 0.05) and the number of dendritic branches (Fig. 4a and c, *p* < 0.05**)** was noted in the neurons been labeled at 7, 14 and 21 days after MCAO surgery compared to time matched sham-operated control. And there is no significant alterations in dendritic phenotypes for the newly generating neurons that born at 28 days after MCAO surgery (Fig. 4a-c, 28 + 14 group, dendritic length: sham, 312.2 ± 15.4 μm vs. MCAO, 275.9 ± 13.56 μm, *p* = 0.0877). These data suggested the deleterious impact of the experience of ischemic stroke on proper maturation of adult newly generated neurons can be lasted for at least three weeks (21 days) after the exposure of hypoxic event.

### The dendritic maturation of the newly generated neurons functionally correlates with the therapeutic benefits of rehabilitation therapy

**Fig. 5:**
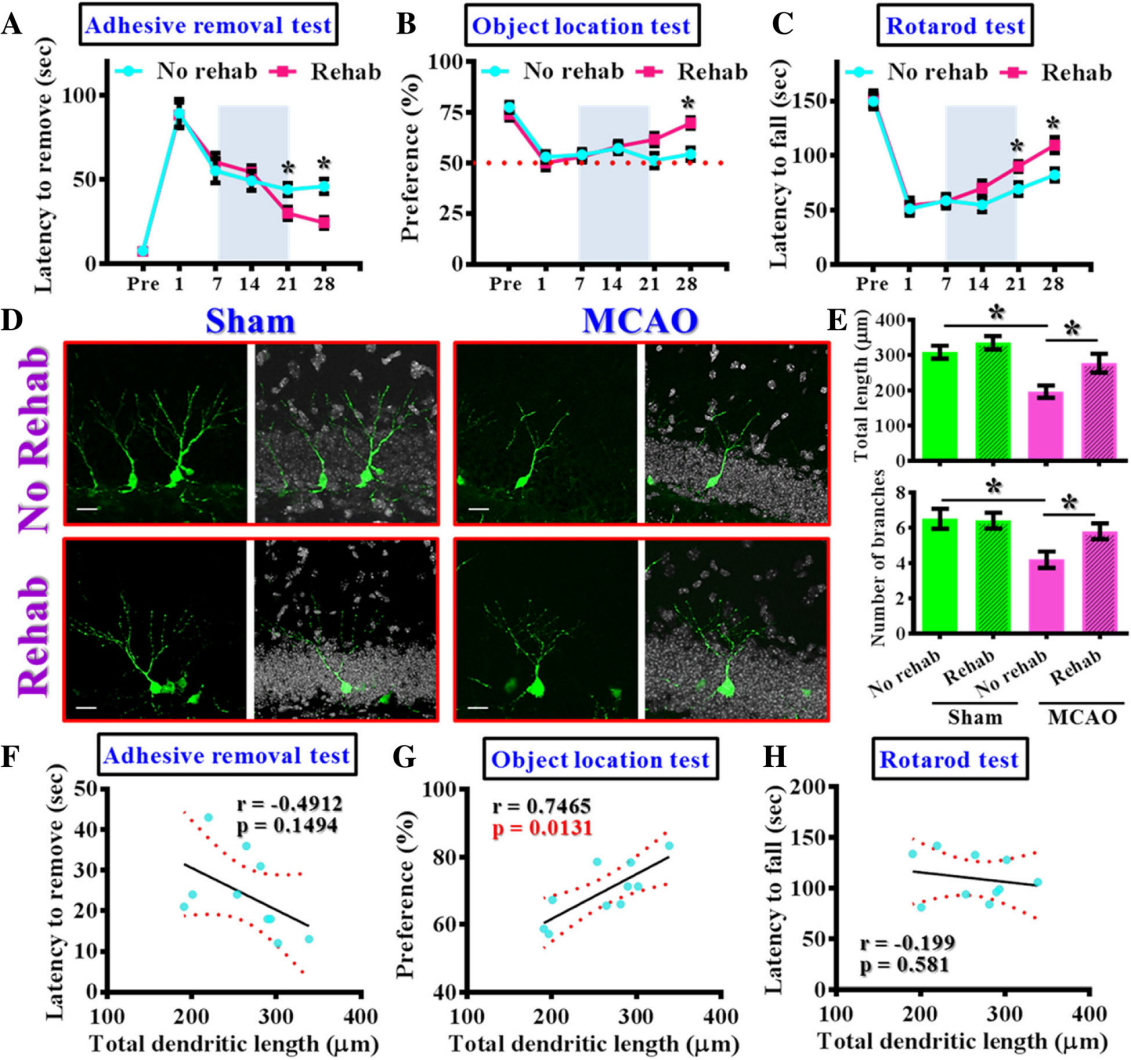
Reversal of aberrant dendritic phenotypes functionally correlates with the rehabilitation therapy induced therapeutic benefits. (**a**) Behavioral performance of adhesive removal task before or at 1, 7, 14, 21 or 28 days after MCAO surgery. Gray area represents the rehabilitation therapy of a period of fourteen days for the Rehab group. Two-way ANOVA analysis showed significant difference between the treatments (*p* < 0.01) and Bonferroni’s post hoc: *p* < 0.05 for the time points of 21 and 28 days. *n* = 10 mice. (**b**) Object location memory test before or days after MCAO surgery. Two-way ANOVA analysis showed significance between the treatments (*p* < 0.01) and Bonferroni’s post hoc: *p* < 0.05 for the time points of 28 days after MCAO. n = 10 mice. (**c**) Rotarod test before or days after MCAO surgery. Two-way ANOVA analysis showed significant difference between the treatments (*p* < 0.001) and Bonferroni’s post hoc: *p* < 0.05 for the time points of 21 and 28 days. *n* = 10 mice. (**d**) Representative images of the retroviral labeled newly generated neurons at 14 dpi from sham-operated or MCAO mice with or without rehabilitation therapy. (**e**) Quantification of total dendritic length and number of branches from 14 dpi newborn neurons. One-way ANOVA for total dendritic length (*F* = 8.629, *p* < 0.001) with Bonferroni’s post hoc analysis. Data are presented as mean ± SEM. *n* = 20 to 40 cells from four mice, * *p* < 0.05. (**f**-**h**) Pearson’s correlation between the total dendritic length averaged from five labeled cells by individual animals with their different neurological function at 28 days after MCAO surgery. *n* = 10 mice

Next, we examined if the rehabilitation therapy, which has long been applied for promoting functional recovery after ischemic stroke both in rodents and clinic [21, 22], might reverse the aberrant dendritic maturation of these newly generated neurons. A significant impairment of multiple neurological function was observed in mice at one day after MCAO surgery, which is evidenced by a prolonged delay to remove the adhesive tape (a sensory and motor related task), impaired in object location memory task (an assay reflecting cognitive function) and poor motor coordination performance (indicator of motor function) on the rotarod task (No rehab group of animals, Fig 5a-c). Such deficits in neurological performance recovered gradually weeks after the hypoxic challenge but still significantly poorer than the pre-stroke levels. Interestingly, a fourteen-day period of rehabilitation therapy (Rehab group of animals, Fig. 5a-c) applied between seven to twenty-one days after MCAO surgery significantly reverse multiple neurological deficits at twenty-eight days after the hypoxic challenge. These data indicated the effectiveness of our rehabilitation treatment.

Then we evaluate the efficacy of rehabilitation therapy in the reversal of aberrant dendritic maturation phenotypes of 14 dpi generating neurons. It is noted the rehabilitation protocol does not significantly alter the total dendritic length or the number of branches in control (sham-operated, Fig. 5d and e) animals. However, the two-week rehabilitating training significantly reversed the dendritic phenotypes after MCAO challenge (Fig. 5d and e, dendritic length: MCAO-no rehab, 196.4 ± 17.14 μm vs. MCAO-Rehab, 279 ± 25.99 μm, *p* = 0.0418). Similar therapeutic benefits were also observed for the changes in the total number of dendritic branches (Fig. 5e, *p* < 0.05).

Next, we analyzed whether normalization of the aberrant dendritic phenotype might be functionally correlated with the recovery-promoting outcome of rehabilitation therapy. By analyzing the total dendritic length from individual animals and correlate with their various parameters of neurological function after rehabilitation therapy, we found there is a trend of correlation between the dendritic phenotype with individual performance in adhesive removal task (Fig. 5f, r = − 0.4912, *p* = 0.1494). And there is a significant positive correlation between the dendritic phenotype with individual cognitive performance (Fig. 5g, *r* = 0.7465, *p* = 0.0131). Meanwhile, there is no significant correlation between the dendritic phenotype with the motor coordination dependent rotarod task (Fig. 5h, *r* = − 0.199, *p* = 0.581). These data indicated that the rehabilitation therapy can phenotypically reverse the aberrant maturation of newly generating neurons in the ischemic brain and its trophic impact on these generating neurons is functionally coupled to their therapeutic benefits in functional recovery of cognitive and partly the sensory function.

Together, our data demonstrate that the normalization of aberrant dendritic maturation of newly generating neurons in the hypoxic brain might functionally participate in the therapeutic benefits of rehabilitation therapy.

### HDAC6 dysfunction pathologically causes aberrant dendritic maturation after hypoxic challenge

Next, we investigate the possible pathological underlying mechanisms contribute to the observed morphological defects by the experience of ischemia. For the importance of microtubule dynamics in the formation of dendritic arborization, we first checked the marker of stabilized microtubule structures: the acetylation levels on Lys40 of α-tubulin after stroke.

**Fig. 6:**
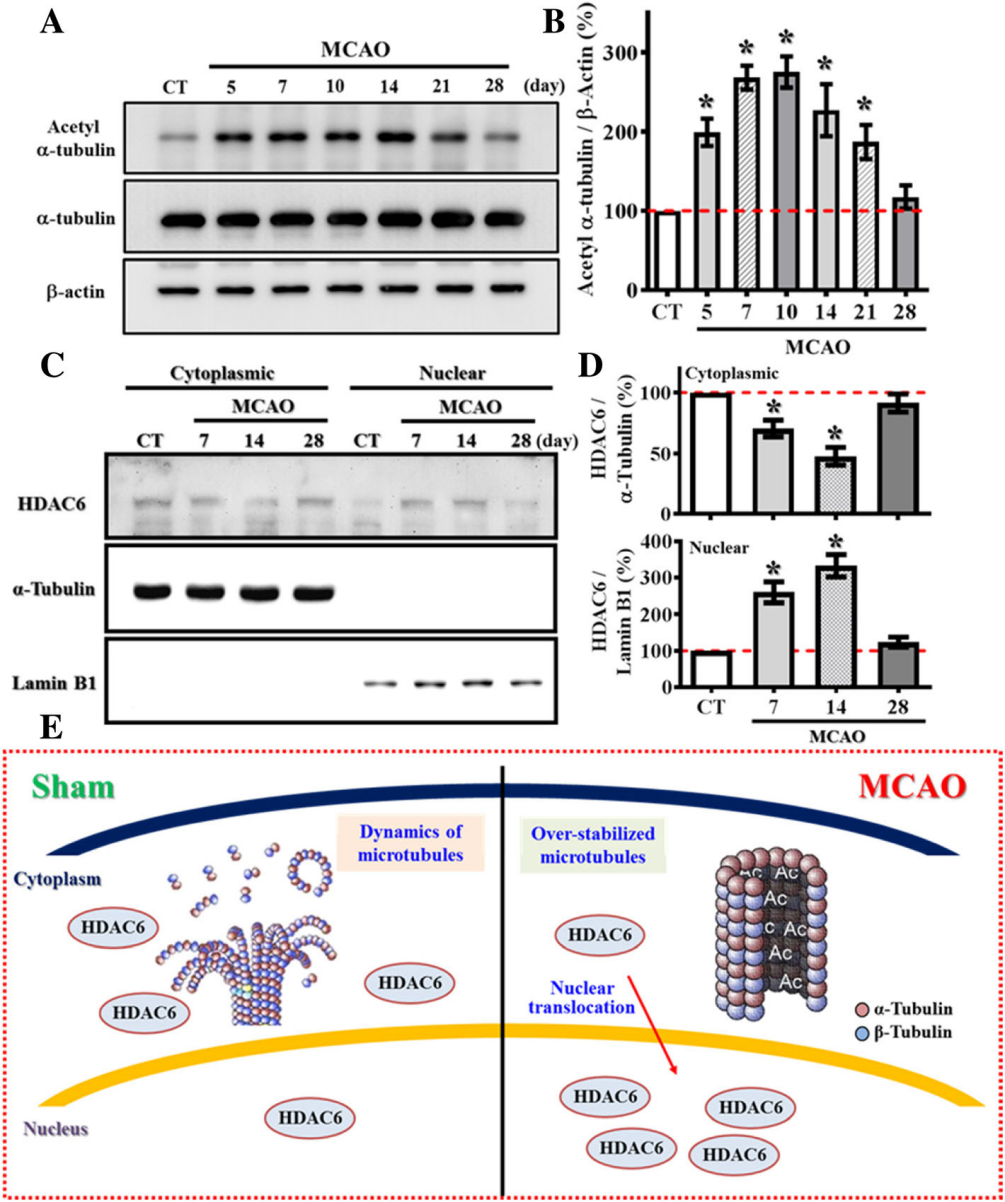
Aberrant nuclear translocation of HDAC6 after stroke causes hyper-acetylation of α-tubulin. (**a**) Representative western blot images of the acetylated-α-tubulin at Lys40, α-tubulin and β-actin levels at different time points after MCAO surgery. (**b**) Quantification of the acetylated-α-tubulin that normalized to β-actin after MCAO surgery. *n* = 4 biological replicates, * *p* < 0.05 compared to sham operated control. (**c**) Representative western blot images of the HDAC6 distributed between cytosolic or nuclear fractions at different time points after MCAO surgery. α-tubulin and Lamin B1 act as the internal control for either cytosolic or nuclear fraction. (**d**) Quantification of the cytoplasmic (top) or nuclear (bottom) protein levels of HDAC6 that normalized to its internal controls at different time points after MCAO. Data are presented as mean ± SEM. *n* = 5, * *p* < 0.05 compared to sham operated control. (**e**) A cartoon model illustrates the aberrant nuclear translocation of HDAC6 after stroke that causes hyper-acetylation α-tubulin

Experimentally, we micro-dissected the granule cell layer of the dentate gyrus where the active neurogenesis take place for the biochemical analyses of samples from different time points after MCAO challenge. We found there is significant increase in acetylation of α-tubulin after stroke (Fig. 6a), and the hyper-acetylation of α-tubulin significantly lasted for at least three weeks after the hypoxic event (Fig. 6b, *p* < 0.05 for the time points of 5, 7, 10, 14 and 21 days after MCAO surgery vs. sham-operated control). Such prolonged increase in the marker of stabilized microtubule correlate with the time course of lasting deleterious impact in dendritic morphology of the newly generating neurons as shown in Fig. 4a-c suggested the potential role of over-stabilized microtubule structure in the pathology of retardation in dendritic maturation of these cells by hypoxic challenge.

HDAC6 is a class IIb deacetylase enzyme that catalyze the de-acetylation of both histone and non-histone protein substrates. HDAC6 is known to function as the major cytoplasmic α-tubulin deacetylase [17], it deacetylates α-tubulin in assembled microtubules that modulate the dynamic flexibility of the microtubules. By analyzing the tissue collected at different time points after MCAO, we found there is no significant different in the protein levels of HDAC6. Since HDAC6 is capable of shuttling between the nucleus and the cytoplasm [23] which might affect modulate its function. We then tested if there might be an aberrant translocation of HDAC6 after the experience of hypoxia challenge; the cytosolic and nuclear fractions were isolated from the micro-dissected granule cell layer of the dentate gyrus at different time points after MCAO surgery. We found the expression of HDAC6 is predominant in cytosol of control animals, but it translocated substantially into the nucleus after stroke (Fig. 6c). Such aberrant nuclear translocation of HDAC6 can be lasted for at least two weeks after the hypoxic event (Fig. 6d, *p* < 0.05 for the time points of 7 and 14 days after MCAO surgery vs. sham-operated control).

Together, the results collected from our biochemical analyses revealed that the aberrant nuclear distribution of HDAC6 after stroke limits its accessibility to its cytosolic substrates, including acetylated α-tubulin. The hyper-acetylation of α-tubulin in turn reduces the dynamic flexibility of microtubules that eventually cause lasting deleterious impact on dendritic maturation of the newly generated neurons (Fig. 6e).

**Fig. 7:**
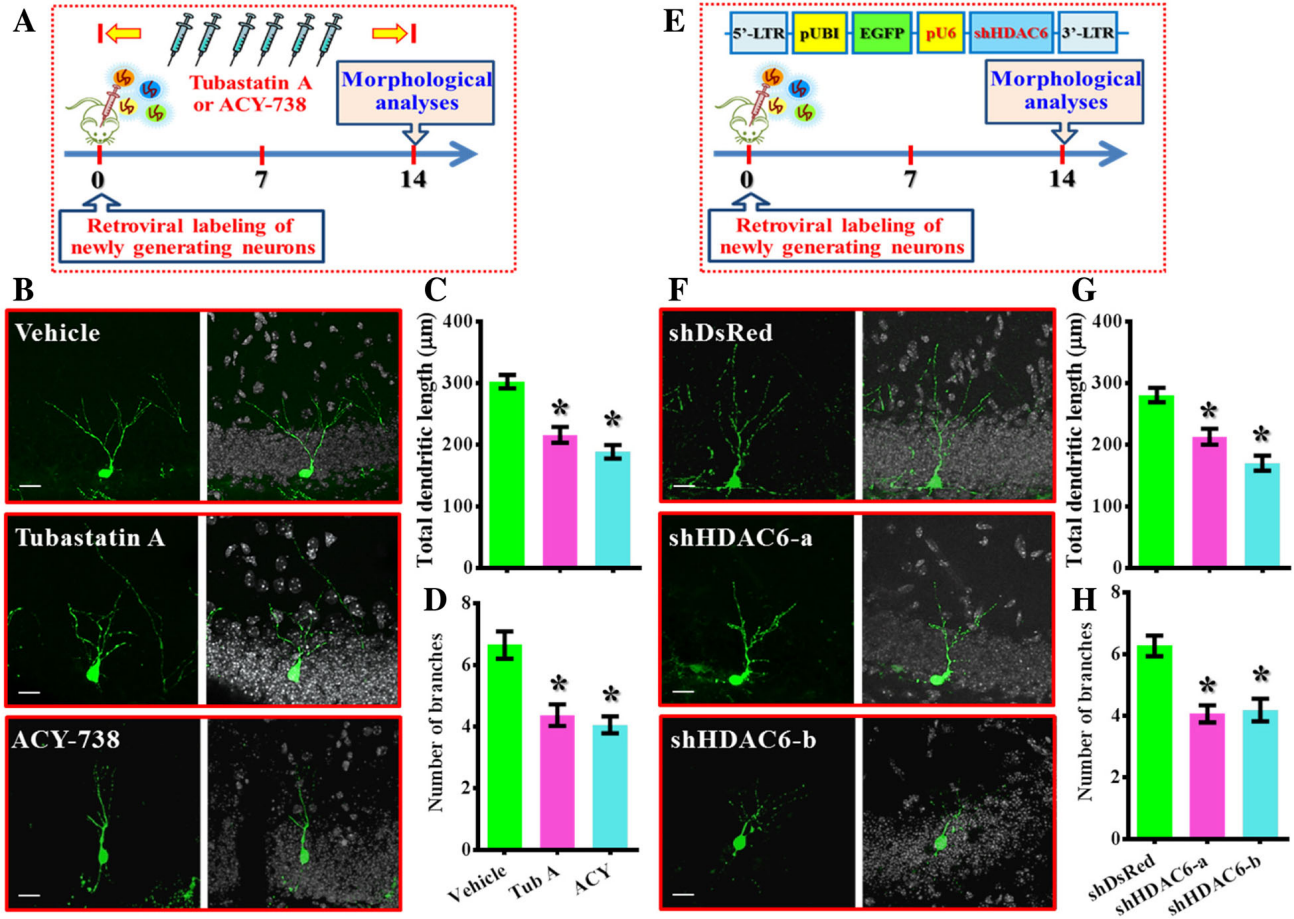
Pharmacological or genetic suppression of HDAC6 phenocopied stroke induced retardation in dendritic maturation. (**a**) Schematic illustration of the procedure for pharmacological suppression of HDAC6 on the dendritic maturation in vivo. Tubastatin A or ACY-738 were applied intraperitoneally every two days. (**b**) Representative images of the retroviral labeled newly generated neurons at 14 dpi. The scale bar is 25 μm. (**c** and **d**) Quantification of total dendritic length and the number of dendritic branches of individual newborn neurons at 14 dpi from different groups. Data are presented as mean ± SEM. *n* = 20 to 40 cells from four mice, data are analyzed by one-way ANOVA with Bonferroni’s post hoc analysis. * *p* < 0.05 compared to vehicle injected control. (**e**) Schematic illustration of the procedure for genetic silencing of HDAC6 on the dendritic maturation in vivo. pU6: U6 promoter, shHDAC6: shRNA targets HDAC6. (**f**) Representative images of the newly generated neurons at 14 dpi. The scale bar is 25 μm. (**g** and **h**) Quantification of total dendritic length and the number of dendritic branches of individual newborn neurons at 14 dpi from different groups. Data are presented as mean ± SEM. *n* = 20 to 40 cells from four mice, data are analyzed by one-way ANOVA with Bonferroni’s post hoc analysis. * *p* < 0.05 compared to shDsRed control

To further strength the functional significance of HDAC6 regulated cellular events in controlling the dendritic maturation of the newly generating neurons, the “mimicry” experiments with either pharmacological or genetic suppression of HDAC6 were designed (Fig. 7a and e). For the pharmacological suppression of HDAC6, retroviruses expressing GFP were stereotaxically injected into the dentate gyrus for labeling of the newly generating neurons in control animals. Then two different HDAC6 inhibitor: Tubastatin A (0.5 mg/kg) or ACY-738 (HDAC6 inhibitor with a higher blood-brain-barrier permeability [24], 5 mg/kg) were intraperitoneal injected every two days. Then, animals were sacrificed at 14 dpi for the analyzing the impact of HDAC6 suppression on the dendritic maturation of the newly generating neurons in vivo. We found there is significant reduction in the total dendritic length (Fig. 7b and c, *p* < 0.05 for the Tubastatin A and ACY-738 group vs. vehicle group) and the number of dendritic branches (Fig. 7b and d, *p* < 0.05) for the newly generating neurons.

Furthermore, to direct confirm the impact of HDAC6 dysfunction affect the maturation of the newly generating neurons in a cell autonomous manner, retroviruses expressing (short-hairpin RNAs) shRNA that genetic silencing of HDAC6 were applied. Experimentally, two HDAC6 shRNA sequences that reduce the expression level of HDAC6 effectively were expressed in retroviral labeled newly generating cells in control animals (Fig. 7e). Then animals were sacrificed at 14 dpi to evaluate the impact of HDAC6 silencing on the dendritic maturation of the newly generating neurons in vivo. We found there is significant reduction in the total dendritic length (Fig. 7f and g, *p* < 0.05 for both shHDAC6 groups vs. shDsRed control) and the number of dendritic branches (Fig. 7f and h, *p* < 0.05) for the newly generating neurons.

Together, these biochemical analyses and in vivo dendritic maturation experiments provide strong evidence indicated the dysfunction of HDAC6 pathologically contributes to the aberrant dendritic maturation phenotypes after hypoxic challenge.

### Proper function of HDAC6 is necessary for rehabilitation therapy induced functional recovery after stroke

**Fig. 8:**
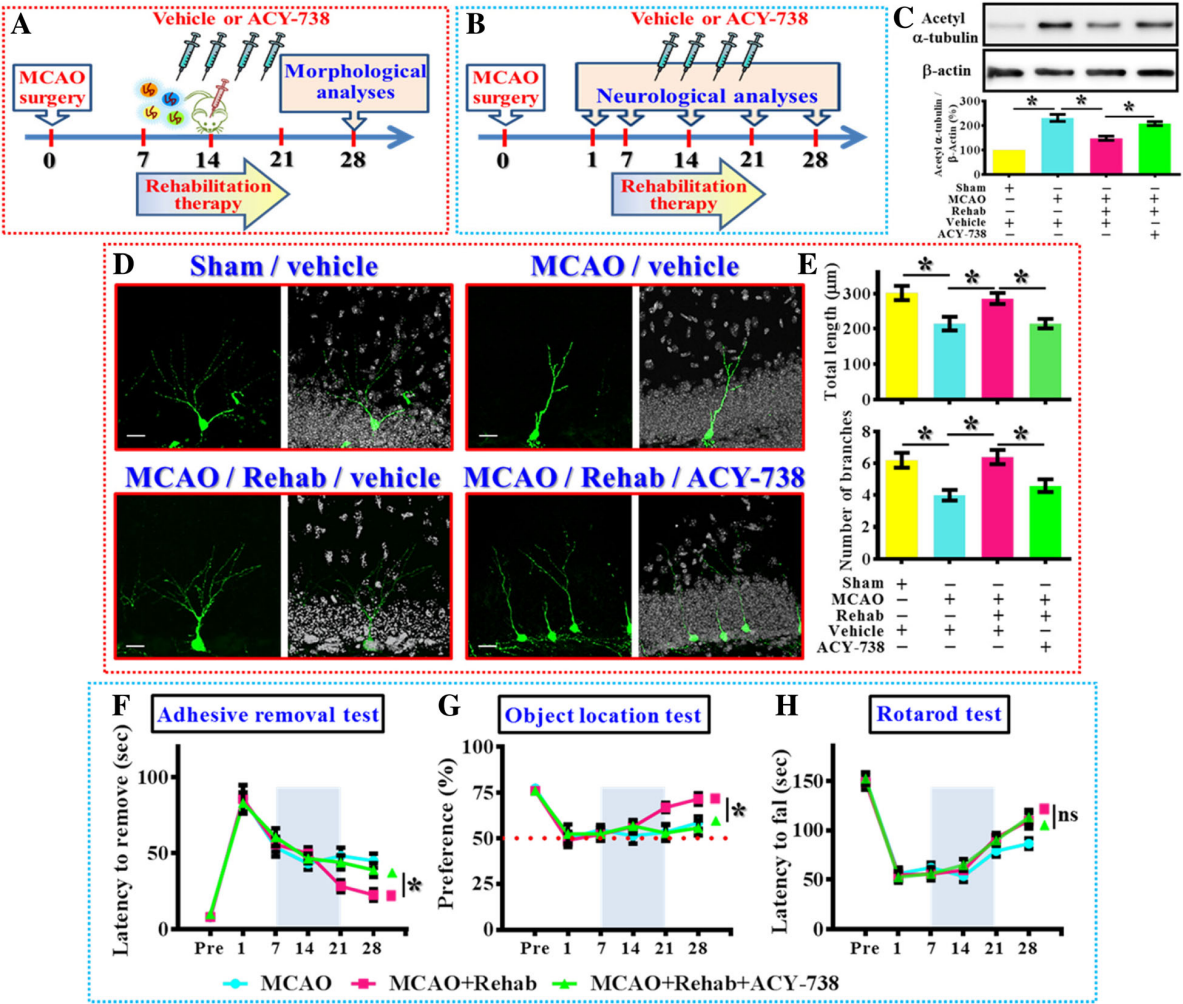
Requirement of HDAC6 in rehabilitation therapy induced functional recovery. (**a**) Schematic illustration the procedure for studying HDAC6 in rehabilitation therapy induced reversal of dendritic phenotypes. (**b**) Schematic illustration the procedure for studying HDAC6 in rehabilitation therapy induced long term functional recovery after MCAO. (**c**) Representative western blot images and quantification of the acetylated-α-tubulin that normalized to β-actin from different experimental groups. *n* = 5 biological replicates, *p < 0.05. (**d**) Representative images of the newly generated neurons for their dendritic elaboration at 14 dpi. “Rehab” indicates the rehabilitation therapy for the mice. The scale bar is 25 μm. (**e**) Quantification of total dendritic length and the number of dendritic branches of individual newborn neurons at 14 dpi from different groups. Data are presented as mean ± SEM. *n* = 20 to 40 cells from four mice, one-way ANOVA with Bonferroni’s post hoc analysis. * *p* < 0.05. (**f**) Behavioral performance of adhesive removal task. Two-way ANOVA analysis showed significant difference between the treatments (*p* < 0.001) and Bonferroni’s post hoc: *p* < 0.05 for the time points of 28 days for the MCAO+Rehab vs. MCAO+Rehab+ACY-738. (**g**) Object location memory test before or days after MCAO. Two-way ANOVA analysis showed significance between the treatments (*p* < 0.01) and Bonferroni’s post hoc: *p* < 0.05 for the time points of 28 days for the MCAO+Rehab vs. MCAO+Rehab+ACY-738. (**h**) Rotarod test before or days after MCAO. Two-way ANOVA analysis showed significant difference between the treatments (*p* < 0.01) and Bonferroni’s post hoc showed no significance for the time points of 28 days for the MCAO+Rehab vs. MCAO+Rehab+ACY-738. *n* = 10 mice

Since we have shown the functional significance of HDAC6 pathologically in modulating the dendritic maturation of newly generating neurons after hypoxic challenge, it should be critical to answer if the proper function of HDAC6 activity for these newly generated neurons is required for rehabilitation therapy induced functional recovery after ischemic stroke (Fig. 8a and b). We first investigated if the function of HDAC6 might be required by rehabilitation therapy induced normalization of aberrant dendritic maturation phenotypes of the newly generating neurons in the hypoxic brain.

Experimentally, retroviral labeling of newly generating neurons were applied at fourteen-days after sham- or MCAO-surgery and animals were sacrificed two weeks after retroviral injection for the morphological analyses of dendritic development of 14 dpi generating cells. Procedures of rehabilitation therapy were applied for two weeks and ACY-738 (5 mg/kg) or vehicle solvent were intraperitoneally injected every two days during the period of rehabilitation therapy to evaluate the influence of HDAC6 activity on the therapeutic benefits of rehabilitation therapy (Fig. 8a). We first confirmed the effect of ACY-738 by evaluating the acetylated levels of α-tubulin, the results indicated that rehabilitation therapy significantly reversed the stroke induced hyper-acetylation of α-tubulin at 14 days after hypoxic challenge (Fig. 8c). However, ACY-738 significantly attenuated the regulation of acetylated α-tubulin by rehabilitation therapy which strongly suggested the involvement of HDAC6 in its effects (Fig. 8c).

And then morphological analyses indicated that rehabilitating training significantly reversed the dendritic phenotypes after MCAO challenge (Fig. 8d and e, dendritic length: MCAO-rehab-vehicle, 285.6 ± 15.4 μm vs. MCAO-Vehicle, 214.7 ± 19.2 μm, *p* < 0.05). However, such therapeutic benefits of rehabilitation therapy on dendritic phonotypes were substantially attenuated by the pharmacological inhibition of HDAC6 (Fig. 8d and e, dendritic length: MCAO-rehab-vehicle vs. MCAO-rehab-ACY-738, 215 ± 13.36 μm, *p* < 0.05).

Furthermore, the role of HDAC6 in rehabilitation therapy induced functional recovery of different neurological function after hypoxic challenge was also evaluated. Experimentally, a two-week period of rehabilitation therapy was applied between seven to twenty-one days after MCAO surgery and ACY-738 (5 mg/kg) or vehicle solvent were intraperitoneally injected every two days during the period of rehabilitation therapy to investigate the role of HDAC6 in functional recovery of the brain (Fig. 8b). We found that rehabilitation therapy significantly reversed multiple neurological deficits at twenty-eight days after the hypoxic challenge; the pharmacological inhibition of HDAC6 significantly attenuated the rehabilitation therapy-induced reversal of multiple neurological deficits (Fig. 8f-h, *p* < 0.05 for both the adhesive removal task and objection location memory task for MCAO-rehab-ACY-738 vs. MCAO-rehab-vehicle at 28 days after MCAO surgery).Together, these morphological and functional analyses provide strong evidence indicated that proper function of HDAC6 is required for rehabilitation therapy induced therapeutic benefits after hypoxic challenge.

## Discussion

Promoting neurogenesis after cerebral ischemic stroke has become a topic of interest, as it is been reported that the adult brain has the capability to generate new neurons [6, 25]. Through the proper functional maturation, these newly generating cells gradually integrated into the existing neural circuitry and actively contribute to critical roles in various neurological functions [5, 26, 27]. Through the retroviral labeling and trace the maturation process of these generating neurons in the post-stroke brain, we provided strong evidence showing that the brain environment is actually not suitable for the proper growing of these cells which might explain the lack of successful clinical outcomes so far [28].

Along with the in vivo findings of the aberrant dendritic phenotypes of newly generating neurons after stroke, our current study has provided some valuable information that may display highly clinical implications for promoting functional recovery of the brain after ischemic stroke. At first, even with a transient stroke protocol for only occluding the blood flow for thirty minutes, we found there is a long lasting (for few weeks) deleterious influence on the structural maturation of the newly generating neurons in the brain. Second, we provide evidence showing that the aberrant dendritic phenotypes can be reversed by rehabilitation therapy and the efficacy of functional recovery of neurological performance correlated with the normalization of dendritic phenotypes, which indicated the two events are functionally coupled. Third, our current study, to the best of our knowledge, is the first report to provide the pathological mechanism that contributes to the impaired maturation of adult neurogenesis after stroke. The aberrant nuclear translocation of HDAC6 contributes to the abnormal dendritic maturation and pathologically limits the functional recovery of the brain. These findings suggested a potential therapeutic strategy that targets HDAC6 for promoting functional recovery toward the patients with stroke in clinic.

In our current study, the retroviral-based single cell labeling approach was applied for the visualization of dynamic changes in dendritic arborization along the neuronal maturation process in vivo. As the subgranular zone (SGZ) of the dentate gyrus is the active neurogenic site in the adult brain and has been shown as a good model system for studying the dedicated maturation process of newly generating neurons in the adult brain [29], retroviruses expressing codon-optimized green fluorescent protein (GFP) were utilized to label and birth-date the newly generating neurons in the adult brain [30, 31]. By taking advantage of the unique properties of retrovirus that target active dividing cells specifically, we are able to precisely trace a definite cohort of adult newly generating neurons and monitor their maturation process at precise time points after they were generated (birth-dating). Meanwhile, we have further optimized the retroviral system with ubiquitin promoter (Fig. 1a) to obtain a more stable and reliable expression of transgenes in the target population of newly generated neurons [32, 33]. Such techniques for morphological studies in vivo, later could be applied for the investigation of other neurodegenerative disorders and help to answer the un-identified pathology for the variety of neurological disorders.

For the rehabilitation therapy, we applied a recently validated rehabilitation protocol for rodents through the combination of task-specific motor rehabilitation therapy with cognitive intensive stimulus of environmental enrichment [34, 35]. Since the therapeutic benefits of neurorehabilitation training is usually task-specific and does not generalize to neurological improvement other than the ones trained, our rehabilitative procedure which is composed of sensory, motor and cognitive stimulation has recently been reported to elicit a synergistic therapeutic effect that promoting functional recovery of multiple neurological function effectively [35]. Based on such rehabilitation-training model that combined with our retroviral labeling of newborn neurons in vivo, we provide direct evidence showing the role of proper maturation of these newly generating neurons is functionally coupled to the therapeutic benefits of rehabilitation therapy.

Our major finding about the proper function of HDAC6 is required for functional recovery of the brain might provide a feasible therapeutic strategy for the unmet need of stroke therapy in clinic. As there is only one drug, the thrombolytic agent: rt-PA that available for the ischemic stroke in clinic. Actually the therapeutic benefit of rt-PA is the removal of thrombus at the acute phase (the golden hour) of ischemic challenge that restores the blood supply of the infarct area. And the transient MCAO stroke model we are using is in fact to mimic the brief interruption of regional blood supply by blood clots and then re-gain the blood flow after the occlusion for thirty minutes. And our data indicated that even a thirty-minute transient loss in regional blood supply (just like one received rt-PA within thirty-minute), there is still a long lasting (for few weeks) functional defeats mechanistically linked to the dysfunction of HDAC6 signaling. These findings suggested that the clinical stroke therapy of rt-PA should combine with the pharmacological approach that normalized the HDAC6 activity which the rt-PA could help to restore the blood supply during the acute phase of stroke challenge and the HDAC6 modulators provide chronic therapeutic benefits that promote functional recovery of the brain afterwards.

Neurons typically develop highly polarized dendritic and axonal morphology along their maturation process that support their functional integration into the complex network of neural circuitry. Accumulating evidence indicated that the dynamic remodeling of microtubules tightly regulates the building of dendritic arborization [15, 36]. As the acetylation levels on Lys40 of α-tubulin is a well-accepted marker of stabilized microtubule structures [16], our current study provide novel findings indicated the aberrant translocation of HDAC6 which results in the hyper-acetylation of α-tubulin and dendritic maturation deficits after hypoxic challenge. There is now an extensive body of evidence demonstrating that HDAC6 may play critical roles in different neurodegenerative diseases such as Huntington’s, Parkinson’s and Alzheimer’s disease [37–39]. However, either protective [40] or negative [24] roles of HDAC6 has been reported under different pathological conditions. Through previous study has indicated that HDAC6 might responsible for the sensing of environmental pathological stimulus and responding with the shuttling between cytoplasm and nucleus that modify the cellular signaling events dynamically between two cellular compartments [41]. However, further detail investigations are required for better understanding of the pathological contribution of HDAC6 in different neurodegenerative disorders.

For the experiments with pharmacological suppression of HDAC6 that ameliorates the beneficial effect of rehabilitation therapy on functional recovery of the brain. Although our data indicated a significant inhibition of rehabilitation therapy induced effect by ACY-738 which supports the conclusion of requirement of HDAC6 in rehabilitation therapy. However, we also noticed that the suppression of HDAC6 only partially inhibits the reversal of dendritic phenotypes and also the recovery of motor function is not significantly affected (Fig. 8). These findings indicated that there might be other molecular mechanisms that could be activated by rehabilitation therapy and contributes to its therapeutic benefits. For example, it has recently been found that α-TAT 1 (or MEC-17) is the major tubulin acetyltransferase in the brain [42, 43] and its stability and expression level decrease significantly by the neuronal injury signal molecules [44]. These previous findings indicated that α-TAT 1 might be downregulated after ischemic stroke and partially contributes to the effect of rehabilitation therapy. Besides, although HDAC6 is the major deacetylation enzyme for α-tubulin, recent evidence indicated that Sirtuin 2 (SIRT2) is also highly expressed in the central nervous system and can function as a deacetylase for α-tubulin as well [45, 46]. Interestingly, a recent study has demonstrated an increase in Sirtuin 2 expression by enriched environment stimulation [47]. Further experiments are required to answer the possible roles of these molecular targets in the effect of rehabilitation therapy.

## Conclusion

The results of this study provide direct evidence demonstrating a lasting loss in structural integrity of newly generating neurons after hypoxic challenge. Meanwhile, the aberrant phenotypes in dendritic maturation correlate with symptomatic impairment of functional recovery of the brain, which is pathologically linked to aberrant nuclear translocation of HDAC6 after stroke. Our current findings might provide a feasible therapeutic strategy that targets HDAC6 for promoting functional recovery toward the patients with stroke in clinic.

## Abbreviations

DAPI: 4′,6-diamidino-2-phenylindole
dpi: days-post-injection
EGFP: enhanced green fluorescent protein
HDAC6: histone deacetylase 6
LTR: Long terminal repeats
MCAO: Middle cerebral artery occlusion
pUBI: Ubiquitin promoter
rt-PA: recombinant tissue plasminogen activator
shRNA: short-hairpin RNA

## References

[1] Jin K, Minami M, Lan JQ, Mao XO, Batteur S, Simon RP (2001). Neurogenesis in dentate subgranular zone and rostral subventricular zone after focal cerebral ischemia in the rat. Proc Natl Acad Sci U S A.

[2] Nakatomi H, Kuriu T, Okabe S, Yamamoto S, Hatano O, Kawahara N (2002). Regeneration of hippocampal pyramidal neurons after ischemic brain injury by recruitment of endogenous neural progenitors. Cell..

[3] Zhang L, Chopp M, Meier DH, Winter S, Wang L, Szalad A (2013). Sonic hedgehog signaling pathway mediates cerebrolysin-improved neurological function after stroke. Stroke..

[4] Ramón y Cajal S, DeFelipe J, Jones EG (1991). Cajal's degeneration and regeneration of the nervous system.

[5] Zhao C, Deng W, Gage FH (2008). Mechanisms and functional implications of adult neurogenesis. Cell..

[6] Gage FH (2002). Neurogenesis in the adult brain. J Neurosci.

[7] Altman J (1969). Autoradiographic and histological studies of postnatal neurogenesis. IV. Cell proliferation and migration in the anterior forebrain, with special reference to persisting neurogenesis in the olfactory bulb. J Comp Neurol.

[8] Aimone JB, Wiles J, Gage FH (2006). Potential role for adult neurogenesis in the encoding of time in new memories. Nat Neurosci.

[9] Ming GL, Song H (2011). Adult neurogenesis in the mammalian brain: significant answers and significant questions. Neuron..

[10] Raber J, Fan Y, Matsumori Y, Liu Z, Weinstein PR, Fike JR (2004). Irradiation attenuates neurogenesis and exacerbates ischemia-induced deficits. Ann Neurol.

[11] Sun C, Sun H, Wu S, Lee CC, Akamatsu Y, Wang RK (2013). Conditional ablation of neuroprogenitor cells in adult mice impedes recovery of poststroke cognitive function and reduces synaptic connectivity in the perforant pathway. J Neurosci.

[12] Cuartero MI, de la Parra J, Perez-Ruiz A, Bravo-Ferrer I, Duran-Laforet V, Garcia-Culebras A, et al. Abolition of aberrant neurogenesis ameliorates cognitive impairment after stroke in mice. J Clin Invest. 2019. 10.1172/JCI120412 PMC6436875 30676325

[13] Woitke F, Ceanga M, Rudolph M, Niv F, Witte OW, Redecker C (2017). Adult hippocampal neurogenesis poststroke: more new granule cells but aberrant morphology and impaired spatial memory. PLoS One.

[14] Niv F, Keiner S, Krishna WOW, Lie DC, Redecker C (2012). Aberrant neurogenesis after stroke: a retroviral cell labeling study. Stroke..

[15] Conde C, Caceres A (2009). Microtubule assembly, organization and dynamics in axons and dendrites. Nat Rev Neurosci.

[16] Hammond JW, Cai DW, Verhey KJ (2008). Tubulin modifications and their cellular functions. Curr Opin Cell Biol.

[17] Hubbert C, Guardiola A, Shao R, Kawaguchi Y, Ito A, Nixon A (2002). HDAC6 is a microtubule-associated deacetylase. Nature..

[18] Sheu JR, Chen ZC, Jayakumar T, Chou DS, Yen TL, Lee HN (2017). A novel indication of platonin, a therapeutic immunomodulating medicine, on neuroprotection against ischemic stroke in mice. Sci Rep.

[19] Wang B, Rao YH, Inoue M, Hao R, Lai CH, Chen D (2014). Microtubule acetylation amplifies p38 kinase signalling and anti-inflammatory IL-10 production. Nat Commun.

[20] Gu Y, Arruda-Carvalho M, Wang J, Janoschka SR, Josselyn SA, Frankland PW (2012). Optical controlling reveals time-dependent roles for adult-born dentate granule cells. Nat Neurosci.

[21] Dobkin BH (2005). Clinical practice. Rehabilitation after stroke. N Engl J Med.

[22] Di Pino G, Pellegrino G, Assenza G, Capone F, Ferreri F, Formica D (2014). Modulation of brain plasticity in stroke: a novel model for neurorehabilitation. Nat Rev Neurol.

[23] Verdel A, Curtet S, Brocard MP, Rousseaux S, Lemercier C, Yoshida M (2000). Active maintenance of mHDA2/mHDAC6 histone-deacetylase in the cytoplasm. Curr Biol.

[24] Guo W, Naujock M, Fumagalli L, Vandoorne T, Baatsen P, Boon R (2017). HDAC6 inhibition reverses axonal transport defects in motor neurons derived from FUS-ALS patients. Nat Commun.

[25] Toda T, Parylak SL, Linker SB, Gage FH (2019). The role of adult hippocampal neurogenesis in brain health and disease. Mol Psychiatry.

[26] Spalding KL, Bergmann O, Alkass K, Bernard S, Salehpour M, Huttner HB (2013). Dynamics of hippocampal neurogenesis in adult humans. Cell..

[27] Ming GL, Song H (2005). Adult neurogenesis in the mammalian central nervous system. Annu Rev Neurosci.

[28] Balami JS, Fricker RA, Chen R (2013). Stem cell therapy for ischaemic stroke: translation from preclinical studies to clinical treatment. CNS Neurol Disord Drug Targets.

[29] Duan X, Chang JH, Ge S, Faulkner RL, Kim JY, Kitabatake Y (2007). Disrupted-in-schizophrenia 1 regulates integration of newly generated neurons in the adult brain. Cell..

[30] Ge S, Goh EL, Sailor KA, Kitabatake Y, Ming GL, Song H (2006). GABA regulates synaptic integration of newly generated neurons in the adult brain. Nature..

[31] van Praag H, Schinder AF, Christie BR, Toni N, Palmer TD, Gage FH (2002). Functional neurogenesis in the adult hippocampus. Nature..

[32] Yang CH, Huang CC, Hsu KS (2012). A critical role for protein tyrosine phosphatase nonreceptor type 5 in determining individual susceptibility to develop stress-related cognitive and morphological changes. J Neurosci.

[33] Kim JY, Liu CY, Zhang FY, Duan X, Wen ZX, Song J (2012). Interplay between DISC1 and GABA signaling regulates neurogenesis in mice and risk for schizophrenia. Cell..

[34] Schmidt A, Wellmann J, Schilling M, Strecker JK, Sommer C, Schabitz WR (2014). Meta-analysis of the efficacy of different training strategies in animal models of ischemic stroke. Stroke..

[35] Jeffers MS, Corbett D (2018). Synergistic effects of enriched environment and task-specific reach training on Poststroke recovery of motor function. Stroke..

[36] Kapitein LC, Hoogenraad CC (2015). Building the neuronal microtubule cytoskeleton. Neuron..

[37] Rivieccio MA, Brochier C, Willis DE, Walker BA, D'Annibale MA, McLaughlin K (2009). HDAC6 is a target for protection and regeneration following injury in the nervous system. Proc Natl Acad Sci U S A.

[38] Dompierre JP, Godin JD, Charrin BC, Cordelieres FP, King SJ, Humbert S (2007). Histone deacetylase 6 inhibition compensates for the transport deficit in Huntington's disease by increasing tubulin acetylation. J Neurosci.

[39] Trushina E., Heldebrant M. P., Perez-Terzic C. M., Bortolon R., Kovtun I. V., Badger J. D., Terzic A., Estevez A., Windebank A. J., Dyer R. B., Yao J., McMurray C. T. (2003). Microtubule destabilization and nuclear entry are sequential steps leading to toxicity in Huntington's disease. Proceedings of the National Academy of Sciences.

[40] Pandey UB, Nie ZP, Batlevi Y, McCray BA, Ritson GP, Nedelsky NB (2007). HDAC6 rescues neurodegeneration and provides an essential link between autophagy and the UPS. Nature..

[41] Sanchez de Diego A, Alonso Guerrero A, Martinez AC, van Wely KH (2014). Dido3-dependent HDAC6 targeting controls cilium size. Nat Commun.

[42] Kalebic N, Sorrentino S, Perlas E, Bolasco G, Martinez C, Heppenstall PA (2013). alphaTAT1 is the major alpha-tubulin acetyltransferase in mice. Nat Commun.

[43] Kim GW, Li L, Ghorbani M, You L, Yang XJ (2013). Mice lacking alpha-tubulin acetyltransferase 1 are viable but display alpha-tubulin acetylation deficiency and dentate gyrus distortion. J Biol Chem.

[44] Wong Victor S. C., Picci Cristina, Swift Michelle, Levinson Max, Willis Dianna, Langley Brett (2018). α-Tubulin Acetyltransferase Is a Novel Target Mediating Neurite Growth Inhibitory Effects of Chondroitin Sulfate Proteoglycans and Myelin-Associated Glycoprotein. eneuro.

[45] Maxwell MM, Tomkinson EM, Nobles J, Wizeman JW, Amore AM, Quinti L (2011). The Sirtuin 2 microtubule deacetylase is an abundant neuronal protein that accumulates in the aging CNS. Hum Mol Genet.

[46] Yuan Q, Zhan L, Zhou QY, Zhang LL, Chen XM, Hu XM (2015). SIRT2 regulates microtubule stabilization in diabetic cardiomyopathy. Eur J Pharmacol.

[47] Grinan-Ferre C, Puigoriol-Illamola D, Palomera-Avalos V, Perez-Caceres D, Companys-Alemany J, Camins A (2016). Environmental enrichment modified epigenetic mechanisms in SAMP8 mouse Hippocampus by reducing oxidative stress and Inflammaging and achieving Neuroprotection. Front Aging Neurosci.

